# Intraoperative Radiation Therapy in Gastric Cancer

**Published:** 2014-06-25

**Authors:** N Bacalbașa, I Bălescu, M Calin, C Balalau

**Affiliations:** * “Carol Davila " University of Medicine and Pharmacy, Bucharest, Romania; ** “Ponderas Hospital" Bucharest, Romania; *** Sf Pantelimon Hospital, Surgery Department, Bucharest

**Keywords:** gastric cancer, local recurrence, intraoperative radiotherapy, prognosis

## Abstract

Abstract

Gastric cancer is one of the most common malignancies worldwide. Although surgery is the only potential curative treatment, the overall survival results remain poor due to the high risks of recurrence, so scientists developed new aggressive adjuvant therapies. That is how the IORT appeared. IORT is a technique designed to provide a large radiation dose to a target tissue considered of being "at risk" to develop recurrence. In this review we resume recent publications which debate the use, efficacy and the overall survival after IORT.

Recurrent tumors may originate from extension of the primary tumor or from regional lymph node metastases not encompassed by the surgical procedure. The appropriate extent of lymph-node dissection continues to be debated. Cuschieri et al demonstrated that there were similar results in 5 years survival and so was the recurrence-free survival between D1 and D2 resection but the proponents of radical lymphadenectomy suggested that prohibitive perioperative mortality and design flaws utilized in some western trials might conceal the survival benefit for radical lymphadenectomy [1-4].

 Nodal involvement and/ or serosal invasion represent major adverse pathological features in patients with primary resectable gastric cancer. In spite of "curative resections", recurrence in locoregional sites represents the most common pattern of failure in patients with gastric cancer with reported rates ranging from 40 to 90%.[**5**] 

 In a series of 107 patients following initial “curative" interventions for gastric cancer, Gunderson et al analyzed the patterns of failure. They reported a rate of 67,3% in local recurrence and suggested that adjuvant radiation therapy should be taken into consideration [**6**]. The figures for local-regional relapse are even higher in the article reported by Wisbeck and al. in a series of 16 necropsies performed in patients formerly operated for resectable gastric cancer, the recurrent loco-regional disease being found in 15 of them. Both reports indicate that loco-regional failure is an almost universal and early event in the natural history of resected gastric cancer [**7**].

 So it is necessary to develop and study additional tumoricidal measures that might eliminate residual malignant tissues, thereby decreasing the risk of recurrence and increasing the overall survival [**8**]. 

 Intraoperative radiotherapy (IORT) is an adjunct technique after surgical excision of solid malignancies which allows the administration of a single dose of radiation to the area with greatest risk of local failure. The radiation target area in the region of the upper abdomen includes potential microscopic tumor extensions [**9**]. In this way, IORT provides the advantage of immediate treatment of local microscopic tumor cell remnants.

 The theoretical advantage of this approach is the ability to deliver a more intensive dose of radiation to the tumor bed while excluding normal tissues from high-dose field [**10**]. This is one of the main advantages of IORT over external beam therapy, which has the limitations imposed by radiosensitive structures partly situated in the radiation field [**9**]. Special linear accelerators have been used for the application of high single-dose radiation to the tumor bed [11,12]. 

 IORT has been known as a feasible radiation treatment since 1907 and has been extensively investigated in Japan since 1964 [7,13] and has been used in several clinical trials in Eastern countries.

 Qin et al established that a single dose of 10-15 Gy is efficient for patients with no clinically detectable lesions, 20 Gy for patients who are suspected to have macroscopic residual lymph nodes, 25 Gy for suspected macroscopic lymph nodes or direct invasion of adjacent structure and 30 Gy for incomplete excision of metastatic lesions [**7**]. In the same time, Abe et al established that single doses of 30-35 Gy could eliminate lymph node metastasis of <3 cm in diameter and a single dose of 28 Gy for undetectable residual lesions [14,15]. 

 In one of his studies, Abe et al evaluated the benefits on survival between patients treated by IORT and those treated by operation alone according to the presence or absence of the serosal invasion and the grade of the lymph node metastasis. The 5-year survival rates for patients who were treated by IORT increased by nearly 10% when the serosal invasion was observed and by nearly 18% when n2 and n3 lymph node metastases were found [**13**]. 

 They also evaluated the benefits of IORT by comparing the results of a group of 115 patients who were treated by intraoperative radiation therapy (IORT) with a control group of patients treated by operation alone. No difference in the survivals of patients with stage I was observed for the two groups. On the other hand, the survivals of patients with stages II through IV who were treated by IORT increased by nearly 10% to 20% in 5 years. A comparative study was also performed .

 In his study, Calvo et al. demonstrated the advantages of intraoperative radiotherapy for the treatment of resectable locally advanced gastric adenocarcinoma. 32 patients with primary gastric adenocarcinoma treated with curative resection (R0) [total gastrectomy (n = 9; 28 %), subtotal (n = 23; 72 %) and D2 lymphadenectomy in all patients] were treated with a component of intraoperative radiotherapy (IORT). The applications concerned the celiac axis and peripancreatic nodal areas. 16 patients also received adjuvant therapy: chemotherapy, external radiotherapy or chemoradiation. After a median follow-up time of 40 months (range, 2–60), locoregional recurrence was observed in 5 (16 %) patients (4 nodal in hepatic hilum and 1 anastomotic). Only pN1 patients developed locoregional relapse. No recurrence was observed in the IORT-treated target volume (celiac trunk and peripancreatic nodes). Overall survival at 5 years was 54.6. Postoperative mortality was 6 % (n = 2) and postoperative complications 19 % (n = 6) [**16**]. 

 Trying to evaluate the efficacy of intraoperative radiotherapy (IORT) followed by concurrent chemotherapy and external beam RT (EBRT) in the treatment of locally advanced gastric adenocarcinoma, Fu et al evaluated a lot of 97 consecutive and non selected patients with newly diagnosed Stage T3, T4, or N+ adenocarcinoma of the stomach underwent gastrectomy with D2 lymph node dissection. After a median follow-up of 24 months, the 3-year locoregional control rate was 77% and 63% in the two groups with or without IORT, respectively. The 3-year overall survival and disease-free survival rate was 47% and 36% in the EBRT group and 56% and 44% in the EBRT+IORT group. They concluded that radical gastrectomy with D2 lymph node dissection and IORT followed by adjuvant chemoradiotherapy appeared to be feasible and well-tolerated in the treatment of locally advanced gastric cancer. The addition of IORT to the modality of treatment significantly improved the 3-year locoregional control rate [**17**]. 

 Hin et al evaluated the proper doses and the outcomes of IORT in 106 patients with stageⅠ-Ⅳ gastric carcinoma who received D2 or D3 radical operation combined with IORT. 77 patients with gastric cancer of the antrum and body underwent distal gastrectomy. After the resection and before alimentary reconstruction, the local region of the hepatoduodenal ligament, the celiac artery and the upper margin of the pancreas or the gastric bed were irradiated. The others 39 patients underwent proximal gastrectomy or total gastrectomy. The sites of irradiation for this group were the upper margin of the pancreas and the regional para-aorta. In the patients with body, cardiac and total stomach cancer, the tail and body of the pancreas were moved up to the right side margin of the abdominal aorta and the superior mesenteric vein and the para-aortic region received irradiation. IORT was administered to the tumor bed and celiac axis at the time of gastric resection in those patients whose tumor appeared transmural. The 5-year survival rates of patients with stage Ⅱ and Ⅲgastric cancers were significantly improved: 100% for stageⅡ and 60.4% for stage Ⅲ compared to 80.6% for stage Ⅱ, 45.1% for stage Ⅲ for surgery alone. The 5-year survival rates of the stages Ⅲ cancer patients receiving D2 resection combined with IORT were markedly improved, while for those receiving D3 radical resection, only the postoperative 3- or 4-year survival rates were improved. The 5-year patients treated by IORT increased by nearly 10% when the serosal invasion was observed, and by nearly 18% when N2 and N3 lymph node metastases were found. There was no difference between the 5-YSR of patients in stageⅠ, and stage Ⅳ in the two groups. The IORT procedure raised the survival of patients with stages Ⅱand Ⅲ cancer from 15% to 20% [**7**]. 

 Coquard et al analyzed the alternative of intraoperative radiation therapy combined with limited lymph node dissection. 

 He proved that IORT combined with limited lymph node dissection may provide a 5 year overall survival similar to that observed after gastrectomy with extended lymph node dissection but with less postoperative mortality [**18**]. 

 Complications

 When IORT is used for gastric cancer, the critical organs to which exposure must be avoided are the duodenum and jejunum. Less than 40% of the pancreas is generally included in the radiation field. Direct shielding of normal pancreatic tissues should be done if they are not at risk of harbouring microscopic disease [**19**]. Acute and late damage to the pancreas are evaluated by measuring the blood glucose level and serum amylase after IORT. Temporary increases in both occurred after IORT but they came back to normal in a week. It is important to notice that mortality and morbidity rates in patients treated with IORT are not excessive when compared with the mortality and morbidity rates reported in the retrospective studies of patients treated by surgery alone [**7**]. 

 Glehen et al did not detect any acute toxicity of IORT [**20**]. 

 The late toxicity is controversial in the literature because of reported cases of enteritis, gastrointestinal bleedings with or without arterioenteric fistulas, vertebral collapses and liver hemangiomas. DeVilla et al reported an arteriodigestive fistula as a complication associated with intraoperative and external beam radiotherapy following surgery for gastric cancer [**21**]. 

 Zhang et al reported similar rates of acute toxicity in IORT compared to EBRT+IORT such as gastrointestinal toxicity - nausea/vomiting, weight loss, dyspepsia and diarrhea in 39% of cases in IORT+EBRT (similar to EBRT - 37%) and hematological toxicity - leucopenia - in 43% of cases IN IORT+EBRT (compared to 45% in EBRT). 

 No anastomotic fistula or wound dehiscence were observed in the IORT group [**22**]. 

 On the contrary, according to Drognitz, complication rates are significantly increased with the use of IORT (44% vs 20%; p<0•05), which should be weighed carefully against the benefits of improved locoregional control. The same author also reported that there are no significant benefits of survival after IORT [**23**]. 

## Conclusions

IORT, as a component of adjuvant radiation therapy treatment, seems to be an efficacious and well tolerated treatment for patients with locally advanced gastric carcinoma, which promotes a better local control rates. IORT as a boost to EBRT can be a method to improve local control rate on coeliac area after gastrectomy [**7,24**]. Escalated radiation doses with concurrent chemotherapy used in an adjuvant setting is a strategy that deserves to be optimized and then evaluated in randomized clinical trials [**22**]. 
